# Recent Progress in Computational and Data Sciences for Additive Manufacturing

**DOI:** 10.3390/ma18051177

**Published:** 2025-03-06

**Authors:** Tuhin Mukherjee, Qianru Wu

**Affiliations:** 1 Department of Mechanical Engineering, Iowa State University, Ames, IA 50011, USA; 2 Jiangsu Key Laboratory of 3D Printing Equipment and Manufacturing, School of Electrical & Automation Engineering, Nanjing Normal University, Nanjing 210023, China; qrwu@nnu.edu.cn

Additive manufacturing (AM), often referred to as 3D printing, is a preferred technique for producing components that are challenging to manufacture through conventional methods. This approach facilitates the direct fabrication of complex parts in a single step from a 3D digital model. Today, AM components are widely utilized across various sectors, including healthcare, aerospace, the automotive industry, energy, the marine sector, and consumer products [1]. Examples of such components include custom medical implants tailored to individual patients, aero-engine parts, intricate geometries with internal channels, lattice structures, and materials designed with location-specific chemical compositions, microstructures, and properties [2]. The materials used include metals, polymers, ceramics, and composites. Among these, metal and alloy printing is advancing most rapidly due to its growing applications, high demand, and ability to produce specialized, functional parts. Various AM techniques are employed based on the material, geometry, and complexity of the desired component [3]. For instance, powder bed fusion (PBF) and directed energy deposition (DED) are commonly utilized for metallic parts. These processes involve melting fine layers of powder or wire feedstocks using high-energy sources like lasers, electron beams, or electric arcs, followed by solidification to form the final part. Similarly, different processes are used in the industry for printing polymers, ceramics, and composite materials.

In AM, a reduction in defects, maintaining geometric consistency, and control of microstructure and mechanical properties cannot be achieved by time-consuming and expensive experimental trials because of the involvement of many variables with a large parameter window. Physics-based computational models are often used as an alternative. Figure 1 provides a few examples of using such models in AM. However, the evolution of microstructures, properties, and defects depends on many complex physical processes, and the mechanistic understanding of many of these processes is not fully developed. The use of data science techniques such as machine learning can automate several steps, including process monitoring, defect detection, sensing, and process control, and can help in the selection of appropriate processing conditions to improve structure and properties. This would minimize the need for human intervention and significantly improve the process efficiency, productivity, and part quality, and reduce materials and energy waste and cost.

Topics in this Special Issue include applications of modeling and machine learning for the novel design of additively manufactured products, additive manufacturing processes, alloy design, tailoring microstructures, customized mechanical and chemical properties, improved creep resistance, fatigue life, and serviceability, reducing defects and residual stresses and distortion. The scope of this Special Issue also includes all AM processes for alloys, ceramics, and polymers.

This Special Issue contains a total of 12 articles including 11 research articles [4,5,6,7,8,9,10,11,12,13,14] and a review paper [15] on the applications of process monitoring, modeling, and statistical analysis in metal additive manufacturing. The 11 research articles uniquely contribute to six distinct areas: (i) the prediction of temperature fields, (ii) keyhole and molten pool geometry calculations, (iii) the estimation of part geometry, (iv) the determination of part surface characteristics, (v) defects and anomaly detection, and (vi) the prediction of mechanical properties, as discussed below.

Sajadi et al. [7] and Fagersand et al. [9] explore computational approaches for predicting temperature fields in metal AM. Sajadi et al. [7] introduce a physics-informed online learning framework for real-time temperature prediction in metal AM using physics-informed neural networks (PINNs). By integrating physics-based inputs and loss functions, the model adapts dynamically to unseen process conditions, demonstrating superior performance in critical regions like the heat-affected zone and melt pool. The approach highlights the role of hyperparameters, such as learning rate and batch size, in optimizing performance for diverse conditions. Fagersand et al. [9] use deep learning, specifically multilayer perceptrons (MLPs), to predict temperature history in wire arc additive manufacturing of aluminum bars. Training on finite element simulations, their models achieve low error rates under baseline conditions but show reduced accuracy for new process parameters, particularly varying scanning speeds. Together, these studies emphasize data- and physics-driven frameworks for the prediction of temperature fields during metal AM.

Wu et al. [5] and Dong et al. [10] use deep learning techniques for predicting molten pool and keyhole geometries. Wu et al. [5] focus on DED, developing surrogate models based on recurrent neural networks (RNNs) like LSTM, Bi-LSTM, and GRU to predict melt pool characteristics. Their models achieve high accuracy, with an R^2^ of 0.98 for peak temperature prediction and over 0.88 for melt pool geometry. Dong et al. [10] investigate laser PBF, using a computer vision tool leveraging the BASNet deep learning model to segment keyhole morphologies from X-ray images. Achieving average accuracies of 91.24% for the keyhole area and 92.81% for the keyhole boundary shape, this tool automates the labeling process, enabling faster and more reliable analysis of keyhole dynamics. Together, these studies identify the potential of deep learning to reliably predict molten pool and keyhole geometries during metal AM.

The article by Hermann et al. [11] introduces a novel decision-making workflow to optimize process parameters for laser DED. Acknowledging the limitations of current analytical, numerical, and machine learning methods in predicting optimal parameters, the authors propose a Gaussian Process Regression (GPR) model. This model predicts the geometry of single DED tracks based on input parameters while incorporating uncertainty quantification (UQ). By leveraging UQ and expert user knowledge, the workflow facilitates the inverse task of identifying parameter sets that minimize deviations between desired and actual track geometries. The GPR model, trained and validated using 379 experimental track cross-sections, demonstrates its efficacy through two illustrative test cases. This approach reduces reliance on trial-and-error experimentation, enabling a more systematic and user-centric method to achieve precise track geometries in laser DED process.

Zhou et al. [6] investigate the use of laser DED for repairing structural aluminum alloys. The study addresses challenges such as uneven material flow and defects caused by unequal powder particle size. A multiscale, multiphysics model integrating discrete element and finite volume methods is developed to analyze the fluid dynamics and thermal behavior of the molten pool during the repair process. Additionally, a macroscale thermomechanical model evaluates stress evolution and verifies the structural integrity of deposited layers. Mengesha et al. [14] explore the surface hardness and scratch resistance of electroless nickel plating on additively manufactured composite components. Using K-means clustering and Taguchi’s design of experiments, the study quantifies scratch widths and evaluates their relationship to hardness levels. Enhanced characterization through SEM imaging improves analysis accuracy, highlighting the potential of Ni-plating for improving surface properties in industrial applications.

Sinha et al. [8] address the challenge of gas porosity in laser PBF. By combining mechanistic modeling and experimental data, the authors propose a dimensionless gas porosity index to predict and mitigate pore formation. Tested against independent data, the index achieves 92% accuracy for alloys like stainless steel 316, Ti-6Al-4V, Inconel 718, and AlSi10Mg, with AlSi10Mg being the most prone to porosity. A gas porosity map is developed for practical process optimization. In contrast, Khan et al. [13] focus on defect detection in metal AM through machine learning and optical tomography (OT). Using layer-wise OT imaging, a Random Forest Classifier identifies anomalies, validated against CT data. The model achieves a 99.98% detection accuracy, correlating with 79.40% of CT-detected defects. Together, these studies advance defect mitigation in AM through mechanistic modeling and machine learning.

The studies by Pazireh et al. [4] and Scime et al. [12] explore data-driven methodologies to predict the mechanical properties of AM parts. Pazireh et al. [4] investigated the effects of toolpath patterns, geometries, and layering on the mechanical properties of DED parts. Using finite element simulations, a linear mixed-effects model, principal component analysis, and self-organizing map clustering, they identify important relationships between process parameters and residual stresses, strains, and mechanical properties. Scime et al. [12] focus on qualifying laser PBF parts using in situ sensor data, integrating powder bed imaging, machine health metrics, and laser paths. Machine learning models trained on over 6000 tensile specimens predict tensile properties with a 61% error reduction compared to non-data-driven approaches. Both studies underscore the importance of leveraging machine learning and data analysis to predict mechanical properties in AM parts.

This Special Issue of “Materials” attracted numerous submissions, and the final publication consists of 12 high-quality peer-reviewed articles. In addition to highlighting exciting advancements, the articles in this Special Issue identified several contemporary scientific, technological, and economic challenges that require immediate attention. They emphasized the need for future research and development to enable the production of high-quality parts in a cost-effective way. It is evident that work in these critical areas of AM, especially focusing on modeling and machine learning, is still in its early stages, and needs further research and development.

## Figures and Tables

**Figure 1:**
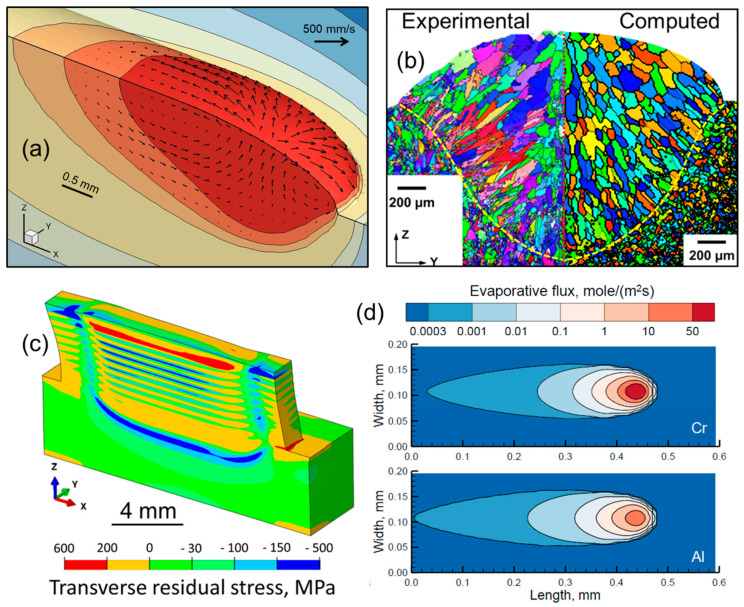
Results from mechanistic models of metal additive manufacturing showing (**a**) 3D temperature and velocity fields during directed energy deposition, (**b**) grain structure during directed energy deposition that matches with experimental data, (**c**) 3D residual stress distribution during directed energy deposition, and (**d**) evaporative flux of alloying elements such as Cr and Al during powder bed fusion. These figures are owned by the authors.
